# Foodborne pathogens

**DOI:** 10.3934/microbiol.2017.3.529

**Published:** 2017-06-29

**Authors:** Thomas Bintsis

**Affiliations:** Department of International Trade, TEI of West Macedonia, Kastoria, Greece

**Keywords:** foodborne pathogens, pathogenic bacteria, pathogenic viruses, pathogenic parasites, foodborne outbreaks

## Abstract

Foodborne pathogens are causing a great number of diseases with significant effects on human health and economy. The characteristics of the most common pathogenic bacteria (*Bacillus cereus*, *Campylobacter jejuni*, *Clostridium botulinum*, *Clostridium perfringens*, *Cronobacter sakazakii*, *Esherichia coli*, *Listeria monocytogenes*, *Salmonella* spp., *Shigella* spp., *Staphylococccus aureus*, *Vibrio* spp. and *Yersinia enterocolitica*), viruses (Hepatitis A and Noroviruses) and parasites (*Cyclospora cayetanensis*, *Toxoplasma gondii* and *Trichinella spiralis*), together with some important outbreaks, are reviewed. Food safety management systems based on to classical hazard-based approach has been proved to be inefficient, and risk-based food safety approach is now suggested from leading researchers and organizations. In this context, a food safety management system should be designed in a way to estimate the risks to human health from food consumption and to identify, select and implement mitigation strategies in order to control and reduce these risks. In addition, the application of suitable food safety education programs for all involved people in the production and consumption of foods is suggested.

## 1. Introduction

The association between the consumption of food and human diseases was recognized very early and it was Hippocrates (460 B.C.) who reported that there is a strong connection between food consumed and human illness [1]. Foodborne pathogens (e.g. viruses, bacteria, parasites) are biological agents that can cause a foodborne illness event. A foodborne disease outbreak is defined as the occurrence of two or more cases of similar illness resulting from the ingestion of a common food [2].

Foodborne illness occurs when a pathogen is ingested with food and establishes itself (and usually multiplies) in the human host, or when a toxigenic pathogens establishes itself in a food product and produces a toxin, which is then ingested by the human host. Thus, foodborne illness is generally classified into: (a) foodborne infection and (b) foodborne intoxication. In foodborne infections, since an incubation period is usually involved, the time from ingestion until symptoms occur is much longer than that of foodborne intoxications.

More than 200 different food-borne diseases have been identified [3]. The most severe cases tend to occur in the very old, in the very young, in those who have compromised immune system function, and in healthy people exposed to a very high dose of an organism [2]. The symptoms, onset of symptoms and the most common responsible microorganisms for the major foodborne illnesses are shown on Table 1.

In the European Union (EU) for the year 2015, 26 member states reported a total of 4,362 food-borne outbreaks, including waterborne outbreaks. Overall, these outbreaks caused 45,874 cases of illness (209 more than 2014), 3,892 hospitalisations (2,546 less than 2014) and 17 deaths (10 less than 2014) [4]. The overall reporting rate of food-borne outbreaks in the EU was 0.95 per 100,000 population, which represents a slight decrease compared with data provided for 2014 [4]. Most of the outbreaks reported in 2015 were caused by bacterial agents (33.7% of all outbreaks), in particular *Salmonella* spp. (21.8% of all outbreaks) and *Campylobacter* spp. (8.9% of all outbreaks), even though the reporting of outbreaks involving these agents has been declining over the recent years. Bacterial toxins ranked second among the causative agents in food- and waterborne outbreaks and were reported in 19.5% of the total outbreaks while viruses, which were the agents most frequently reported in 2014, accounted for 9.2% of total outbreaks in 2015 [4]. Parasites and other causative agents, in particular histamine, were reported in less than 3% of the outbreaks. Furthermore, for a third of the reported outbreaks (34%) the causative agent remained unknown [4].

The implicated food vehicles were mostly of animal origin, in particular eggs and egg products and pig meat (both accounting for 10% of all strong-evidence outbreaks), broiler meat (9%) and cheese (8%) followed by fish and fish products (7%), milk and dairy products (5%), bovine meat (4%) and crustaceans (3%) [4]. In 2015, *Salmonella* spp. in eggs was associated with the highest number of reported foodborne outbreaks and was among the top-5 food-pathogen combinations in terms of the overall number of cases of illness and hospitalisations in outbreaks. However, the number of reported outbreaks caused by *Salmonella* spp. and associated with the consumption of “eggs and egg products” has been decreasing in the last 5 years [4]. Household was by far the most frequent place of exposure. In strong-evidence foodborne outbreaks, *Salmonella* spp. was the most common agent reported in private households, whereas, “bacterial toxins other than *Clostridium botulinum* toxins”, viruses and other causative agents were more frequently reported in public settings such as canteens, workplace catering, restaurants and pubs [4].

The characteristics of the most important foodborne pathogens, the illnesses they cause, together with some of the most important outbreaks they have been implicated are studied in this review.

**Table 1.** Symptoms, onset of symptoms and responsible microorganisms or toxin for the major foodborne illnesses.

Approximate onset time to symptoms	Predominant symptoms	Associated organism or toxin
1–7 h, mean 2–4 h	Nausea, vomiting, retching, diarrhea, abdominal pain, prostration	*Staphylococcus aureus* and its enterotoxins
8–16 h (2–4 h if emesis predominant)	Vomiting or diarrhea, depending on whether diarrheic or emetic toxin present; abdominal cramps; nausea	*Bacillus cereus* (emetic toxin)
12–48 h	Nausea, vomiting, watery non-bloody diarrhea, dehydration	Norovirus
2–36 h (mean 6–12 h)	Abdominal cramps, diarrhea, putrefactive diarrhea (*Cl. perfringens*), sometimes nausea and vomiting	*Clostridium perfringens*
6–96 h (usually 1–3 days)	Fever, abdominal cramps, diarrhea, vomiting, headache	*Salmonella* spp., *Shigella* spp., *E. coli*
6 h to 5 days	Abdominal cramps, diarrhea, vomiting, fever, malaise, nausea, headache, dehydration	*Vibrio cholearae* (O1 and non-O1), *Vibrio parahaemolyticus*
1–10 (median 3–4) days	Diarrhea (often bloody), abdominal pain, nausea, vomiting, malaise, fever (uncommon with *E. coli* O157:H7)	Enterohaemorrhagic *E. coli*, *Campylobacter* spp.
3–5 days	Fever, vomiting, watery non-inflammatory diarrhea	Rotavirus, Astrovirus, enteric Adenovirus
3–7 days	Fever, diarrhea, abdominal pain	*Yersinia enterocolitica*
1 to several weeks	Abdominal pain, diarrhea, constipation, headache, drowsiness, ulcers, variable—often asymptomatic	*Entamoeda histolytica*
3–6 months	Nervousness, insomnia, hunger pains, anorexia, weight loss, abdominal pain, sometimes gastroenteritis	*Taenia saginata*, *Taenia solium*
2 h to 6 days, usually 12–36 h	Vertigo, double or blurred vision, loss or light reflex, difficulty in swallowing, dry mouth, weakness, respiratory paralysis	*Clostridium botulinum* and its neurotoxins
4–28 days	Gastroenteritis, fever, oedema around eyes, perspiration, muscular pain, chills, prostration, laboured breathing	*Trichinella spiralis*
7–28 days	Malaise, headache, fever, fever, cough, nausea, vomiting, constipation, abdominal pain, chills, rose spots, bloody stools	*Salmonella Tympi*
10–13 days	Fever, headache, myalgia, rash	*Toxoplasma gondii*
Varying periods	Fever, chills, headache, arthalgia, prostration, malaise, swollen lymph nodes and other specific symptoms of disease in question	*Listeria monocytogenes*, *Campylobacter jejuni*

After: [5],[6].

## 2. Foodborne Bacteria

Bacteria are the most common cause of foodborne diseases and exist in a variety of shapes, types and properties. Some pathogenic bacteria are capable of spore formation and thus, highly heat-resistant (e.g. *Clostridium botulinum, C. perfringens, Bacillus subtilus, Bacillus cereus*) [7]. Some are capable of producing heat-resistant toxins (e.g. *Staphylococcus aureus, Clostridium botulinum*). Most pathogens are mesophilic with optimal growth temperature range from 20 °C to 45 °C. However, certain foodborne pathogens (i.e. psychrotrophs), such as *Listeria monocytogenes*, and *Yersinia enterocolitica* are capable of growth under refrigerated conditions or temperatures less than 10 °C [7].

### 2.1. Bacillus cereus

*Bacillus cereus* are members of the family *Bacillaceae*; they are Gram-positive, motile rods, and they have the ability to form spores [7]. Most *Bacillus* spp. are found throughout the environment, including soils, fresh and marine water environments. Spores produced by *B. cereus* possess appendages and/or pili and are more hydrophobic than any other *Bacillus* spores. These properties enable the spores to adhere to many different types of surfaces and to resist removal during cleaning and sanitation [8]. Vegetative cells of *B. cereus* grow at temperatures ranging from 4–15 to 35–55 °C but prefer 30–40 °C, depending on the strain [9]. The organism grows at pH 4.9–9.3, but the inhibitory effect of pH is reduced in foods as evidenced by limited growth on meat at pH 4.35 [5],[7] The minimum *a_w_*, for growth has been established at 0.93, but it has been suggested to use 0.912 as the minimum required for growth, because fried rice tends to have *a_w_* values ranging from 0.912 to 0.961 and readily supports *B. cereus* growth [8],[10].

*B. cereus* produces two types of toxins, the emetic (vomiting) and the diarrhoeal one, causing two types of illness. The emetic syndrome is caused by emetic toxin produced by the bacteria during the growth phase in the food. The diarrhoeal syndrome is caused by diarrhoeal toxins produced during growth of the bacteria in the small intestine. The rapid onset of the emetic type is characterized by nausea and vomiting while the late onset of the diarrheal type is characterized by diarrhea and abdominal pain. Both syndromes (i.e., diarrheal and emetic) are a result of *B. cereus* endospores surviving the cooking process, after which germination and subsequent proliferation of vegetative cells occurs at some point during storage. Foods that are frequently implicated in *B. cereus* diarrheic food poisoning include meat products, soups, vegetables, puddings, sauces, milk and milk products [8]. Symptoms are characterized by abdominal pain, nausea, and diarrhea after an incubation period of approximately 8–16 h (Table 1). Diarrheal syndrome symptoms generally persist no longer than 12–24 h. After a 1–5 h incubation period, emetic syndrome symptoms include primarily nausea and vomiting and persist for 6–24 h (Table 1). Foods implicated in *B. cereus* emetic food poisoning include fried and cooked rice, pasta, noodles, and pastry [8]. The diarrheal syndrome type of food poisoning results from the action of a thermolabile enterotoxic complex, whereas the emetic syndrome type involves the action of a thermostable toxin.

Due to the formation of adhesive endospores, *B. cereus* is commonly present in food production environments and then spreading to all kinds of foods. They produce a range of virulence factors that may cause unpleasant disease in humans when present in food or the gastrointestinal tract and it is one of the major foodborne pathogenic bacteria, although in most cases disease is mild and of short duration [11].

Genetic and genomic analyses have revealed that *B. cereus* is very similar to *Bacillus anthracis* and that some strains have plasmids resembling the toxin plasmids of *Bacillus anthracis*. 310 genomes have been completed up to now according to the data retrieved from NCBI, 2017. The median total length of the genome is 5.6626 Mb [12].

*B. cereus* related food poisoning is not a notifiable disease in most countries; therefore, incidence data is limited. It is recognized that there may be significant under reporting of *B. cereus* illness due to the generally mild, short duration and self-limiting symptoms, however, fatal incidences have been reported [11]. It is estimated that *B. cereus* caused 0.7% of foodborne illness among the 31 major pathogens in the US [13],[14].

In a study for *B. cereus* outbreaks [15] were most often attributed to rice dishes (50%); fried rice was the most common type of rice dish (68%). Rice dishes were most commonly cooked and served immediately (42%) or were part of large, solid masses of food (33%) [15]. Twenty-four percent of *B. cereus* outbreaks were associated with meat or poultry dishes. Meat or poultry dishes were cooked and served immediately (50%), roasted (33%), or part of liquid or semisolid mixtures (17%) [15].

In an outbreak at a birthday party in Bari, Italy, the characteristics were consistent with available reports on foodborne outbreaks caused by *B. cereus* [16]. The short incubation period and the predominance of vomiting suggested an emetic toxin. The distribution of cases by time of onset suggested a common source of contamination by a bacteria or a toxin. *B. cereus* was isolated from basmati rice and fecal specimens. Poor food handling and storage was most probably the cause of the outbreak [16].

At a college sport day in Thailand, 470 individuals were ill with vomiting, nausea, and abdominal pain; approximately half of the individuals reported diarrhea [17]. The ingestion of cream-filled eclairs was significantly associated with illness and the mean incubation period was 3.2 hours, which suggested a preformed toxin in the food; initial laboratory investigation indicated presence of *B. cereus* [17]. *B. cereus* was reported as a major causative agent of foodborne illness in the Netherlands in 2006 (causing 5.4% of the foodborne outbreaks) and in Norway in 2000 (causing 32% of foodborne outbreaks) [17]. Pasta salad and spaghetti leftovers were the cause of two outbreaks, where the clinical data and the rapid onset of symptoms, together with the microbiological and molecular study, pointed to *B. cereus* as the causative agent [18],[19].

### 2.2. Campylobacter jejuni

*Campylobacter* spp. are members of the family *Campylobacteriaceae* and *Campylobacter* *jejuni* is one of the most common causes of diarrheal illness. *C. jejuni* is responsible for approximately 850,000 illnesses, 8,500 hospitalizations, and 76 deaths in the US each year [13]. The World Health Organization (WHO) estimates that ∼1% of the population of Western Europe will be infected with campylobacters each year [20]. Extensively found throughout nature, *C. jejuni* can colonize the intestines of both mammals and birds, and transmission to humans occurs via contaminated food products. This organism can invade the epithelial layer by first attaching to epithelial cells, then penetrating through them. Diarrhea results from damage to the epithelial cells. Systemic infections can also occur causing more severe illnesses [12]. 932 genomes have been completed up to now according to the data retrieved from NCBI. The median total length of the genome is 1.686 Mb [12].

*Campylobacter* spp. are small (0.2–0.9 µm wide and 0.2–5.0 µm long), spiral formed, Gram-negative bacteria with 18 species, six sub-species and two biovars [20]. *Campylobacter* genomes are relatively unstable; several mechanisms that may lead to this genetic instability have been proposed, including bacteriophage activity, DNA recombination and transformation [5]. They are very different from other pathogens associated with foodborne disease in that they are essentially microaerophilic, growing best in an atmosphere containing approximately 10% CO_2_ and approximately 5% O_2_. The species pathogenic for man also have a rather narrow temperature range for growth with a maximum temperature of ∼46 °C and a minimum of 30 °C. These are classified as thermophilic campylobacters [20].

In 2015, Campylobacter continued to be the most commonly reported gastrointestinal bacterial pathogen in humans in the EU and has been so since 2005 [4]. The number of reported confirmed cases of human campylobacteriosis was 229,213, a 5.8% decrease compared with the rate in 2014 [4].

*Campylobacter* spp. are part of the normal intestinal flora of a wide variety of healthy domestic and wild animals, including cattle, sheep, goats, pigs, chickens, ducks, geese, wild birds, dogs, cats, rodents, and marine mammals, and are often found associated with bodies of water such as water troughs and streams. Most cases of campylobacteriosis are associated with eating raw or undercooked poultry meat, unpasteurized milk, contaminated water, or from cross-contamination of other foods by these items. All animals used for food can be campylobacter-positive as can many companion species (domestic pets). Samples from the natural environment, such as groundwater, will also frequently contain these pathogens [21]. Ready-to-eat fresh produce contaminated with enteric pathogens presents a risk to consumers. However, its importance as a source of campylobacters is unclear. The number of documented foodborne outbreaks associated with raw fruits, vegetables and unpasteurised fruit juices has increased. Such foods can present a campylobacteriosis risk to public health as a consequence of using contaminated irrigation or washing water.

When stressed, campylobacters enter a “viable but non-culturable state”, characterized by uptake of amino acids and maintenance of an intact outer membrane but inability to grow on selective media; such organisms, however, can be transmitted to animals [22],[23].

In June 2012, 44 persons who attended a wedding reception in Sweden became ill [24]. The outbreak investigation identified chicken liver pâtè as the suspected source of the infection; the liver pâtè had been deliberately undercooked, lightly fried to keep the right texture and mixed with spices [24]. Several *Campylobacter* spp. outbreaks associated with consumption of poultry liver pâtè have been described, especially in the UK [25]–[29], but also in other countries such as Australia [24] and US [30].

A serious outbreak of *Campylobacter* spp. was associated with the consumption of raw milk [31]. *C. jejuni* was isolated in 50 of 88 raw milk samples in New Zealand after a gastrointestinal illness among children in two different camp sites [31]. A drink prepared with raw milk was associated with an outbreak of *C. jejuni* enteritis involving more than 500 participants in a jogging rally in Switzerland, with an attack rate of over 75%. An outbreak of *C. jejuni* enteritis followed the consumption of unpasteurized milk at an attack rate of around 50%; there were cases in all age groups, with the highest number in the 1 to 10 year old group [31]. *C. coli* was isolated from a 9-year-old British boy with persistent diarrhea, whose family had consumed raw goat's milk from a local farm. *C. jejuni* and *E. coli* were found in the feces of goats from the farm, and *C. jejuni* was identified in samples of bulk milk [31].

In October 2013, public health authorities in Australia were notified of a suspected outbreak of gastroenteritis in students and guests following a catered function at a university residential college; a total of 56 cases of gastroenteritis, including seven laboratory-confirmed cases of *C. jejuni* infection, were identified in 235 eligible respondents [32]. *C. jejuni* diversity in epidemiologically related human and food isolates recovered during outbreaks linked to poultry liver [32].

### 2.3. Clostridium botulinum

*Clostridium* spp. are spore-forming bacteria, members of the family *Bacillaceae* and includes obligately anaerobic or aerotolerant, sporeforming rods that do not form spores in the presence of air and, at least in early stages of growth, are usually Gram-positive. In most species, vegetative cells appear as straight or curved rods, varying from short coccoid rods to long filamentous forms with rounded, tapered, or blunt ends, that occur singly, in pairs, or in various chain lengths [7]. Clostridia are found throughout the environment but are most prevalent in the soil and in the intestinal tract of animals. The characteristic shape of clostridia is attributed to the presence of endospores that develop under conditions unfavorable for vegetative growth and distend single cells terminally or sub-terminally [7]. The endospores of many species are extremely sturdy and survive extended boiling in water and exposure to air. Spores germinate under conditions favorable for vegetative growth, such as anaerobiosis and presence of organic substrates [7].

*Cl. botulinum* are motile by means of peritrichous flagella and produce botulinum neurotoxins, the most lethal poison known. There are seven types of botulinum neurotoxin, A through G, based on the antigenic specificity of the toxin produced by each strain [5]. Types A, B, E, and F causing botulism in humans, types C and D causing botulism in birds and mammals, and type G, which has yet to be clearly implicated in a botulism case [5],[7]. Thermal processing is the most common method used to produce shelf-stable, low-acid, moist foods by inactivating *Cl. botulinum* spores.

From the evolutionary perspective, clostridia are considered to be the most ancient bacteria. It is believed that present day *Mollicutes* (*Eubacteria*) have evolved regressively (i.e., by genome reduction) from gram-positive clostridia-like ancestors with a low GC content in DNA. Several species of clostridia (e.g., *Cl. perfringens*, *Cl. botulinum*, *Cl. tetani*) are known opportunistic toxin-producing pathogens in animals and humans [12]. Some species are capable of producing organic solvents (acetone, ethanol, etc.), molecular hydrogen and other useful compounds. There are also species that can fix molecular nitrogen and thus are important participants in biological turnaround of nitrogen compounds in nature. The most common and widely distributed are strains and serovars of *Cl. botulinum* that produce type A toxin. This toxin finds its use in various applications requiring neuroparalitic intervention, including cosmetology (Botox®). 177 genomes have been completed up to now according to the data retrieved from NCBI. The median total length of the genome is 3.898 Mb [12].

*Cl. botulinum* is present in soils, freshwater, marine sediments, and the intestinal tracts of animals. Food sources commonly sampled include primarily honey, which should not be fed to infants less than 1 year of age, as well as fish, meats, vegetables, and infant foods. A variety of foods, such as canned corn, peppers, green beans, soups, beets, asparagus, mushrooms, ripe olives, spinach, tuna fish, chicken and chicken livers, liver pate, luncheon meats, ham, sausage, stuffed eggplant, lobster, and smoked and salted fish have been associated with botulinum toxin [5].

Traditionally, foodborne botulism has been associated with underprocessed and abused sausages or home canned foods; however, in recent years botulism has been acquired through the consumption of contaminated foods such as potato salad, sauteed onions, garlic sauce, cheese, yogurt, bean paste, and olives.

Symptoms of botulinum neurotoxin ingestion appear 12–36 h after consumption of contaminated food and initially may include nausea and vomiting (Table 1). However, these symptoms are followed by the more characteristic neurological signs including visual impairment and acute flaccid paralysis that begins with the muscles of the face, head, and pharynx, descending to involve muscles of the thorax and extremities and leading to possible death from respiratory failure caused by upper airway or diaphragm paralysis [7]. The minimum toxic dose of *Cl. botulinum* neurotoxin has not been determined, but from a human health and food safety standpoint, there should be no tolerance either for the neurotoxin itself or for conditions allowing growth of the organism in foods [7].

Botulinum neurotoxin is synthesized during cellular growth and is subsequently released during cell lysis, where proteolytic cleavage activates the molecule [7]. There are four categories of botulism, which include the classic foodborne botulism derived from the ingestion of preformed toxin in foods, wound botulism resulting from toxin production after organism growth in an infected wound, infant botulism from toxin elaboration in the intestinal tract of infants, and botulism due to intestinal colonization in older children and adults with intestinal disorders or complications resulting in a lack of microbial competition [7]. Botulinum neurotoxin introduced in any of these categories is transported via the bloodstream to neuromuscular junctions, where the toxin irreversibly binds to receptors on peripheral nerve endings and subsequently is internalized into the nerve cell [7].

Recent developments in whole genome sequencing have made a substantial contribution to understanding the genomes, neurotoxins and biology of *Cl. botulinum* Group I (proteolytic *Cl. botulinum*) and *Cl. botulinum* Group II (non-proteolytic *Cl. botulinum*). Two different approaches were used to study genomics in these bacteria; comparative whole genome microarrays and direct comparison of complete genome DNA sequences [33].

The failure to effectively apply the botulinum cook (121 °C for 3 min) to canned or bottled foods has led to many outbreaks of foodborne botulism associated with *Cl. botulinum* Group I. For example, a large outbreak in Thailand in 2006 (209 cases) was associated with consumption of inadequately home-canned bamboo shoots [33]. Inadequate thermal processing of cans of a commercial hot dog chilli sauce in 2007 in US was associated with eight botulism cases, and initially led to the recall of 39 million cans, then an expanded recall of 111 million cans [34]. Temperature abuse of foods intended to be stored chilled has also been responsible for several severe outbreaks of foodborne botulism, including those associated with commercial chilled carrot juice [35] and commercial chicken enchiladas [33],[36].

### 2.4. Clostridium perfringens

*Clostridium perfringens*, previously known as *Clostridium welchii*, belongs to the family *Bacillaceae* and is an important cause of foodborne disease. They are nonmotile, encapsulated rod-shaped cells that produce protein toxins and form spores resistant to various environmental stresses such as radiation, desiccation, and heat [7]. Vegetative cells grow at temperatures ranging from 6 to 50 °C but prefer an optimum temperature between 43 and 47 °C. Growth requires a minimum *a_w_*, of 0.93, a sodium chloride concentration less than 5–8% depending on the strain, and a pH of 5.0–9.0, although 6.0–7.2 is preferred [7],[10].

*Cl. perfringens* are the most prevalent *Clostridium* species found in human clinical specimens, excluding faeces, and has been implicated in simple wound infections to myonecrosis, clostridial cellulitis, intra-abdominal sepsis, gangrenous cholecystitis, postabortion infection, intravascular hemolysis, bacteremia, pneumonia, thoracic and subdural empyema, and brain abscesses [7],[8]. The spores and cells of the organism are frequently associated with dust contamination on many surfaces, including foods such as meat and shellfish, as a result of its ubiquity throughout the environment [7].

*Cl. perfringens* are estimated to be the second most common bacterial causes of foodborne illness in the US, causing one million illnesses each year [37]. Local, state, and territorial health departments voluntarily report *Cl. perfringens* outbreaks to the US CDC through the Foodborne Disease Outbreak Surveillance System. From 1998 to 2010, 289 confirmed outbreaks of *Cl*. *perfringens* illness were reported with 15,208 illnesses, 83 hospitalizations, and eight deaths [37]. The number of outbreaks reported each year ranged from 16 to 31 with no apparent trend over time [37]. The annual number of outbreak-associated illnesses ranged from 359 to 2,173, and the median outbreak size was 24 illnesses [37]. Restaurants (43%) were the most common setting of food preparation; other settings included catering facility (19%), private home (16%), prison or jail (11%), and other (10%) [37]. Among the 144 (50%) outbreaks attributed to a single food commodity, beef was the most common commodity (66 outbreaks, 46%), followed by poultry (43 outbreaks, 30%), and pork (23 outbreaks, 16%) [37]. Outbreaks caused by *Cl*. *perfringens* occur regularly, are often large, and can cause substantial morbidity yet are preventable if contamination of raw meat and poultry products is prevented at the farm or slaughterhouse or, after contamination, if these products are properly handled and prepared, particularly in restaurants and catering facilities [37].

Foodborne illness almost always is a result of temperature abuse, and in many instances, the food vehicle has been improperly cooked meat or meat product that has been left to cook and/or cool too slowly or has undergone insufficient reheating, allowing surviving spores to germinate leading to vegetative cell proliferation. After ingestion and an incubation period of 7–30 h, symptoms typically include cramping and abdominal pain, although nausea and vomiting may also ensue, persisting for 24–48 h [7].

Five toxin-producing types of *Cl. perfringens* have been identified (A through E), and all produce an alpha-toxin (phospholipase) that plays a role in myonecrosis [7]. Type B strains produce beta- and epsilon-toxins, type D strains also produce epsilon-toxin, and type E strains produce an iota-toxin [7]. Almost all reported cases of foodborne gastroenteritis in the US that involve *Cl. perfringens* are a result of type A infection after the ingestion of highly contaminated foods with greater than 10^6^–10^7^ viable vegetative cells, which undergo sporulation in the small intestine and produce enterotoxin [7]. The enterotoxin produced during sporulation is released with the spores during cell lysis. After release, the enterotoxin binds to epithelial cells, causing cytotoxic cell membrane damage and subsequent alteration of permeability, leading to diarrhea and abdominal cramping [7].

An outbreak of *Cl. perfringens* occurred in a care home and fifteen residents reported illness. The likely cause was consumption of mince and vegetable pie and/or gravy [38]. There were a number of issues with food served, in particular the mince products had been cooked, cooled, reheated and served again over a period of several days; fecal sampling revealed the presence of *Cl. perfringens* enterotoxin gene and four samples were indistinguishable by fluorescent amplified fragment length polymorphism, indicating a likely common source [38]. The operator of the home was charged with three offences under the General Food Regulations 2004 and the Food Hygiene (England) Regulations 2006 and was convicted on all counts [38]. Epidemiological evidence can be used to help prosecute businesses with food safety offences in such circumstances [38].

### 2.5. Cronobacter sakazakii

The genus *Cronobacter* consists of a diverse group of Gram-negative bacilli and comprises seven species: *Cronobacter sakazakii*, *Cronobacter malonaticus*, *Cronobacter muytjensii*, *Cronobacter turicensis*, *Cronobacter dublinensis*, *Cronobacter universalis*, and *Cronobacter condimenti* [39].

Among these, *Cr. sakazakii*, formerly *Enterobacter sakazakii*, is associated with infant septicemia, meningitis, and necrotizing enterocolitis. Originally isolated from powdered formula, it has also been shown to compartmentalize cerebral ventricles and cause brain abcesses in neonates. This species produces a yellow pigment when grown at 30 °C, but this fades at 37 °C [12]. 49 genomes have been completed up to now according to the data retrieved from NCBI. The median total length of the genome is 4.5475 Mb [12].

Infections in elderly and immunocompromised adults have also been reported [40], and the epidemiology of these cases suggests that other potential sources of contamination exist, such as the home environment [41] and retail foods (e.g. dried milk powder, dried meats, legumes, nuts, dried flours and spices) [42]. Although *Cronobacter* spp. have been detected in this wide assortment of foods, only contaminated powdered infant formula has been linked epidemiologically with infant infections and outbreaks caused by *Cr. sakazakii* [43]. The source of this contamination is thought to be powdered infant formula manufactured under poor Good Manufacturing Practice (GMP); however, extrinsic contamination of opened cans and human carriage may also be possible [39].

### 2.6. Escherichia coli

*Escherichia coli* is a Gram-negative, non-spore forming rod. It may or may not be mobile; some rods are flagellated and some are not [43]. The organism is a facultative anaerobe and ferments simple sugars such as glucose to form lactic, acetic, and formic acids; the optimum pH for growth is 6.0 to 8.0; however, growth can occur as low as pH 4.3 and as high as 9 to 10 pH [43].

*E. coli* comprise a large and diverse group of bacteria. Most strains of *E. coli* are harmless; other strains have acquired characteristics, such as the production of toxins, which make them pathogenic to humans [44]. 5351 genomes have been completed up to now according to the data retrieved from NCBI. The median total length of the genome is 5.171 Mb [12]. Pathogenic variants of *E. coli* (pathovars or pathotypes) cause much morbidity and mortality worldwide; many of these pathotypes are a major public health concern as they have low infectious doses and are transmitted through ubiquitous mediums, including food and water [45]. Transmission of *E. coli* occurs when food or water that is contaminated with feces of infected humans or animals is consumed. Contamination of animal products often occurs during the slaughter and processing of animals [44]. The use of manure from cattle or other animals as fertilizer for agricultural crops can contaminate produce and irrigation water [44]. *E. coli* can survive for long periods in the environment and can proliferate in vegetables and other foods.

Pathogenic *E. coli* have been categorized into six groups according to the pathogenic mechanism: (1) Enteropathogenic *E. coli* (EPEC); (2) Enterohemorrhagic *E. coli* (EHEC, also known as Shiga toxin—producing *E. coli* [STEC] and formerly referred to as verotoxin-producing *E. coli* [VTEC]); (3) Enterotoxigenic *E. coli* (ETEC); (4) Enteroaggregative *E. coli* (EAggEC); (5) Enteroinvasive *E. coli* (EIEC); and (6) Attaching and Effacing *E. coli* (A/EEC) [44],[45].

STEC infection can cause episodes of mild to severe diarrhea, and 5–10% of infections develop into Hemolytic Uremic Syndrome (HUS)—a severe complication marked by profuse bleeding that can lead to kidney failure and death. STEC strain O157:H7 is estimated to cause 63,000 illnesses, 2,100 hospitalizations, and 20 deaths each year [13]. The principal reservoir for this zoonotic pathogen is the intestinal tract of cattle, but other animals may also serve as reservoirs. O157:H7 emerged as a significant public health threat in 1982 during two outbreaks of disease that investigators associated with the consumption of undercooked ground meat [13]. A wide variety of foods, including fresh produce, have since served as a vehicle for *E. coli* O157:H7 outbreaks. Food producers must report the presence of *E. coli* O157:H7 to health authorities [13].

In 1982, an investigation by the CDC of two outbreaks of severe bloody diarrhea, associated with the same fast-food restaurant chain, led to the identification of a strain of *E. coli*, one that expressed O-antigen 157 and H-antigen 7, that had not previously been recognized as a pathogen [46]. Subsequently, this strain was shown to belong to a category of *E. coli* that produce toxins which are similar to Shiga toxin of *Shigella dysenteriae* and distinct from previously described *E. coli* heat-stable and heat-labile toxins. As data were accumulating on the role of *E. coli* O157:H7 as a pathogen, parallel work in Canada was uncovering high rates of infection with this and other Shiga toxin-producing *E. coli* in patients with the HUS [44]. Subsequent research has indicated that *E. coli* O157:H7 is the cause of 85–95% of cases of hemolytic uremic syndrome in North America, and that non-O157 Shiga toxin-producing *E. coli* are responsible for another 5–15% [47].

In 2015, 5,901 confirmed cases of STEC infections were reported in the EU [4]. The EU notification rate was 1.27 cases per 100,000 population, which was slightly lower than the notification rate in 2014. The EU notification rate following the large outbreak in 2011 was higher in 2012–2015 than before the outbreak but stabilised in the last 2 years in 2014–2015 [4]. In 2011, a rare strain of *E. coli* O104:H4 caused the second largest and the deadliest outbreak of *E. coli-*associated disease ever recorded. Between May 21 and July 22, 2011, more than 4,000 people became ill in 16 countries, and 50 individuals died [48]. By the time the outbreak ended in early July, 2011, there were reports of more than 4,000 illnesses, 800 cases of HUS, and 50 deaths in Germany and 15 other countries [49]. The outbreak was unusual because of the high proportion of adult patients (∼25%) with HUS and the frequent development of neurological symptoms in these patients [50]. Research suggests that these clinical characteristics were due to the unique combination of traits carried by the pathogen, which included features typical of enteroaggregative *E. coli* and the capacity to produce Shiga toxin [50]. This strain also has a distinct set of additional virulence and antibiotic-resistance factors [50]. In addition, eight deaths due to STEC infection were reported in the EU which resulted in an EU case fatality of 0.2% among the 3,352 confirmed cases for which this information was provided. As in previous years, the most commonly reported STEC serogroup in 2015 was O157 (41.7%), although its relative proportion compared to other serogroups declined. This is possibly an effect of increased awareness and of more laboratories testing for other serogroups. Serogroup O157 was followed by serogroups O26, O103, O91, O145, O146 and O128.

The proportion of non-typable STEC strains continued to increase in 2015 [4]. Since a 1993 outbreak associated with hamburgers purchased from a fast food chain resulted in more than 500 laboratory-confirmed infections with *E. coli* O157:H7 and at least 4 deaths [51], several interventions have been introduced to reduce the contamination of beef during processing and in the retail and restaurant industries [52].

In 2006, investigators linked at least 183 illnesses and one death to the consumption of fresh spinach contaminated with *E. coli* O157:H7 [53]. Among the ill persons, 95 (52%) were hospitalized and 29 (16%) had HUS [53]. In response to the growing outbreak—which included cases across 26 states and Canada—FDA advised consumers to stop eating all uncooked, fresh spinach, or products containing uncooked spinach [53]. Epidemiological studies traced the contamination to a single shift at a Natural Selections Foods processing plant in San Juan Batista, California, which had produced 42,000 bags of pre-washed and ready-to-eat baby spinach [54]. Based on isolates from contaminated produce from sick consumers, investigators matched the outbreak strain to environmental samples from a single field of organic spinach in central California [55]. Environmental sampling revealed the presence of the outbreak strain in river water and the feces of cattle and wild pigs less than 1 mile away from the spinach field [55],[56].

Interestingly, for the outbreak in Germany, investigators initially identified fresh produce—including leafy greens, tomatoes, and cucumbers as likely sources [57]. Traceback studies of disease clusters in five German provinces that were affected early in the outbreak pointed to sprouts produced by an organic grower in Lower Saxony [58]. A smaller, second wave of illnesses around the French city of Bordeaux also resulted from the consumption of sprouts, and patient isolates from both outbreaks were identical [59]. It was later discovered that sprout seeds associated with both outbreaks had a common origin in a 16.5 tons shipment of fenugreek seeds from Egypt [59]. Upon the shipment's arrival in Germany in 2009, various distributors in Germany and other European countries subdivided, packaged, repackaged, and widely distributed these seeds as part of thousands of packets of “seed mixes” [59]. Despite extensive recall efforts, the complex chain of packaging and distribution may mean that contaminated seeds could remain on store shelves until their expiration date in 2014 [59]. The pathogen was not isolated from any remaining batches of the suspect seeds, and questions remain as to the source and reservoir of the contaminating pathogen [60].

Flour and flour-based mixes have been suspected or implicated as the source of other foodborne *Salmonella* and STEC O157 outbreaks [61],[62]. Evidence obtained at one restaurant showed that dessert pizzas were made with the same dough mix used in traditional pizzas, but used thicker dough and might have been undercooked at some locations [62]. On May 31, 2016, General Mills recalled several sizes and varieties of flours due to possible E. coli contamination; in June 2016, laboratory testing by FDA isolated STEC O121 in open samples of General Mills flour collected from the homes of ill people in Arizona, Colorado, and Oklahoma [62]. This outbreak is a reminder that is it not safe to taste or eat raw dough or batter; flour or other ingredients used to make raw dough or batter can be contaminated with STEC and other germs that can make people sick [62].

### 2.7. Listeria monocytogenes

*Listeria monocytogenes* is one of the leading causes of death from food-borne pathogens especially in pregnant women, newborns, the elderly, and immuno-compromised individuals [63]. Infections in pregnant women can be devastating to the fetus, resulting in miscarriages, stillbirths, and birth defects [63]. It is found in environments such as decaying vegetable matter, sewage, water, and soil, and it can survive extremes of both temperatures (1–45 °C) and salt concentration marking it as an extremely dangerous food-born pathogen, especially on food that is not reheated and is carried asymptomatically by numerous animal species. The bacterium has been found in a variety of raw foods, such as uncooked meats and vegetables, as well as in foods that become contaminated after cooking or processing. It can spread from the site of infection in the intestines to the central nervous system and the fetal-placental unit. It can cause meningitis (inflammation of the membrane surrounding spinal cord and brain), gastroenteritis (inflammation of mucous membranes of stomach and intestine), and septicemia (systemic spread of bacteria and toxins in the blood) can result from infection [63]. It has 13 serotypes, including 1/2a, 1/2b, 1/2c, 3a, 3b, 3c, 4a, 4ab, 4b, 4c, 4d, 4e, and 7; among them, serotypes 1/2a, 1/2b, and 4b have been associated with the vast majority of foodborne infections [5]. 1243 genomes have been completed up to now according to the data retrieved from NCBI. The median total length of the genome is 2.974 Mb [12].

Listeriosis is a serious infection usually caused by eating food contaminated with *L. monocytogenes.* Although it is a relatively rare disease with a high mortality rate (20–30 %) that makes it one of the deadliest food-borne threats [64]. Unlike many other foodborne pathogens, *Listeria* multiplies in cold environments such as refrigerators [65]. It can quickly spread in damp buildings, dripping off pipes or ceilings onto food. Once *Listeria* bacteria get into a food-processing factory, they can live there for years, sometimes contaminating food products [64],[65].

In the EU for the year 2015, 28 member states reported 2,206 confirmed human cases of listeriosis [4]. The EU notification rate was 0.46 cases per 100,000 population, which was similar to 2014 [4]. There was a statistically significant increasing trend of listeriosis over 2008–2015; nineteen member states reported 270 deaths due to listeriosis in 2015, which was the highest annual number of deaths reported since 2008 [4]. The EU case fatality was 17.7% among the 1,524 confirmed cases with known outcome [4]. Listeriosis infections were most commonly reported in the elderly population in the age group over 64 years old and particularly in the age group over 84 years [4].

It is estimated that *L. monocytogenes* causes on average 1,591 episodes of domestically acquired food-borne illnesses, 1,455 hospitalizations, and 255 deaths annually in the US [13]. Over the last 10 to 15 years, increasing evidence suggests that persistence of *L. monocytogenes* in food processing plants for years or even decades is an important factor in the transmission of this foodborne pathogen and the root cause of a number of human listeriosis outbreaks. *L. monocytogenes* persistence in other food-associated environments (e.g., farms and retail establishments) may also contribute to food contamination and transmission of the pathogen to humans [13].

Although the available data clearly indicate that *L. monocytogenes* persistence at various stages of the food chain contributes to contamination of finished products, continued efforts to quantitatively integrate data on *L. monocytogenes* persistence (e.g., meta-analysis or quantitative microbial risk assessment) will be needed to advance our understanding of persistence of this pathogen and its economic and public health impacts [66].

Whole apples have not been previously implicated in outbreaks of foodborne bacterial illness. A nationwide listeriosis outbreak associated with caramel apples was investigated [67]. Outbreak-associated cases were compared with non-outbreak-associated cases and environmental investigations were performed; 35 outbreak-associated cases were identified in 12 states; 34 (97%) were hospitalized and seven (20%) died [67]. This outbreak highlights the importance of minimizing produce contamination with *L. monocytogenes*; investigators should perform single-interviewer open-ended interviews when a food is not readily identified [67].

*L. monocytogenes* is killed by pasteurization and cooking; however, in some Ready-To-Eat (RTE) foods contamination may occur after factory cooking but before packaging. RTE foods pose higher risk for listeriosis as they are ingested without any further processing, such as cooking, that would kill *L. monocytogenes*. Many of these foods use refrigeration, among other methods, to restrict bacterial growth during their shelf-life. While these standard practices work well for most bacteria, they are not adequate for Listeria control as the organism is capable of growth at refrigeration temperature and is often tolerant to freezing temperature, high salt and low pH [68]. RTE products, such as delicatessen (deli) meats and soft cheeses have repeatedly been identified by foodborne disease control programs as sources of outbreaks and products that put humans at risk for listeriosis. Although, most listeriosis cases tend to be sporadic in occurrence, outbreaks do occur frequently. Due to the global phenomenon of outbreaks associated with Listeria in deli meats and cheese, it requires an urgent attention from national and international authorities through rigorous procedures for its identification, surveillance procedures that can bring more awareness to the general public [68].

One of the largest and deadliest multi-state outbreaks of listeriosis in the US occurred in late summer of 2011. The incident marked the first time that *Listeria* spp. contamination had been linked to whole cantaloupe and one of the few times it had been linked to fresh produce [69]. 146 Individuals had become ill after being infected with the outbreak strain of listeria; 29 deaths and 1 miscarriage had also been attributed to the infection [69]. In response to the CDC outbreak investigation, the cantaloupe producer, announced a voluntary recall of the 300,000 cases of cantaloupes harvested and produced between July and September [69]. The recall included 1.5 to 4.5 million melons that were distributed at supermarkets and chain stores in at least 28 states [69]. FDA inspectors cited unsanitary conditions—such as old, corroded, and difficult-to-clean equipment and standing pools of water—and the absence of processing steps to cool the melons before cold storage as likely contributors to contamination [69].

### 2.8. *Salmonella* spp.

This group of *Enterobactericiae* have pathogenic characteristics and are one of the most common causes of enteric infections (food poisoning) worldwide. They were named after the scientist Dr. Daniel Salmon who isolated the first organism, *Salmonella choleraesuis*, from the intestine of a pig [7]. The genus *Salmonella* is divided into two species that can cause illness in humans: *S. enterica and S. bongori* [5]. *Salmonella* is further subdivided into serotypes, based on the Kaufmann-White typing scheme first published in 1934, which differentiates *Salmonella* strains by their surface and flagellar antigenic properties. *Salmonella* spp. are commonly referred to by their serotype names. For example, *Salmonella enterica* subsp. *enterica* is further divided into numerous serotypes, including *S.* Enteritidis and *S.* Typhimurium [5]. Certain serovars of *Salmonella enterica* are responsible for more serious diseases such as Typhoid fever. The presence of several pathogenicity islands (PAIs) that encode various virulence factors allows *Salmonella* spp. to colonize and infect host organisms. There are two important PAIs, *Salmonella* pathogenicity island 1 and 2 (SPI-1 and SPI-2) that encode two different type III secretion systems for the delivery of effector molecules into the host cell that result in internalization of the bacteria which then leads to systemic spread. 5323 *Salmonella enterica* genomes have been completed up to now according to the data retrieved from NCBI. The median total length of the genome is 4.783 Mb [12].

*Salmonella* spp. are the leading bacterial causes of food-borne illness in the US [13]. The CDC estimates that more than 1 million people in the US contract *Salmonella* each year, with an average of 19,000 hospitalizations and 380 deaths [13]. *Salmonella* spp. live in the intestines of most livestock and many wild animals. *Salmonella* spp. infection usually occurs when a person eats food contaminated with the feces of animals or humans carrying the bacteria. *Salmonella* outbreaks are commonly associated with eggs, meat, and poultry, but these bacteria can also contaminate other foods such as fruits and vegetables. More recently, the CDC has reported a total of 258 persons infected with the outbreak strain of *Salmonella* Bareilly (247 persons) or *Salmonella* Nchanga (11 persons) from 24 states and the District of Columbia [70]. Thirty-two ill persons have been hospitalized, and no deaths have been reported. Collaborative investigation efforts of state, local, and federal public health agencies indicate that a frozen raw yellow fin tuna product, known as Nakaochi Scrape, from Moon Marine USA Corporation is the likely source of this outbreak [70].

In EU for the year 2015, a total of 94,625 confirmed salmonellosis cases (126 fatal) were reported by 28 member states, resulting in an EU notification rate of 21.2 cases per 100,000 population. This represented a 1.9% increase in the EU notification rate compared with 2014. There was a statistically significant decreasing trend of salmonellosis in the 8-year period between 2008 and 2015 [4]. As in previous years, the two most commonly reported *Salmonella* serovars in 2014 were *S.* Enteritidis and *S.* Typhimurium, representing 45.7% and 15.8%, respectively, of all reported serovars in confirmed human cases. Cases of *Salmonella* Infantis, the fourth most common serovar continued to decrease in 2015. Cases of *Salmonella* Stanley still remained, as in the last 2 years, at a higher level than before the large outbreak reported in 2011–2012 [4].

In 1994, 138,000 gallons of ice cream were contaminated by *Salmonella*. This “single batch” of ice cream was consumed by individuals in 15 states, where it sickened an estimated 225,000 individuals [71]. *Salmonella* spp. contamination of peanuts and peanut products led to one of the largest product recalls in US history. More than 714 people in 46 states were sickened in this outbreak and 9 individuals died [72]. Investigators traced the contamination to a single facility that produced peanuts, peanut butter, and peanut paste; more than 200 companies used these foodstuffs as ingredients in a variety of other products, such as brownie products, cake and pie products, candy products, cereal products, cookie products, cracker products, prepackaged meals, snack mix products, ice cream, pet food, and topping products [72]. The recall extended to more than 3,900 products [72],[73]. In 2008, 1,450 individuals in 43 states and the District of Columbia became ill from salmonellosis and two patients died after consuming jalapeño and serrano peppers imported from Mexico; investigations traced the contaminated peppers to one farm in Mexico, but the source of contamination is unknown [73].

Pathogens may be passively internalized during produce processing, and this occurred in 1999, when mangoes imported to the US from Brazil were treated to kill possible Mediterranean fruit fly by dipping them in hot water, after which they were chilled in a cold-water bath [74]. The cold water was not treated, it was not potable and it was contaminated with *Salmonella* Newport, which infected 78 people in 13 states [74],[75]. During 1973–2011, of the 1,965 outbreaks of salmonella where a food vehicle was implicated, 96 were attributed to beef, accounting for 3,684 illnesses [76].

In EU between 2014 and 2015, a total of 162 cases, mostly from France, followed by Belgium, the Netherlands, Spain, Denmark and Sweden were reported, including 86 (53%) women [77]. Using whole genome sequencing (WGS), the cause was identified as *Salmonella enterica* serotype Chester; *S.* Chester was more likely to have eaten in a restaurant and visited the coast of Morocco [77]. Outbreaks associated with *S.* Chester have been reported: in Australia, associated with sea turtle meat in 1998 and with tap water in 2005; in the US, associated with frozen meals (cheesy chicken and rice) in 2010 and in Canada, associated with headcheese in 2010 [78]–[81].

### 2.9. *Shigella* spp.

The genus *Shigella* is a member of the family Enterobacteriaceae and possesses four serogroups that have been traditionally treated as species: serogroup A as *Shigella dysenteriae*, serogroup B as *Shigella flexneri*,serogroup C as *Shigella* *boydii*, and, serogroup D as *Shigella sonnei.* Whereas serogroups A, B, and C consist of 38 serotypes, serogroup D possesses only one [7]. *Shigella* are non-motile, non-spore-forming, facultative anaerobic Gram-negative rods. They can grow at temperatures ranging from 6 to 48 °C, but prefer 37 °C, and *S.* *sonnei* appears to be able to tolerate lower temperatures better than the other serogroups. Optimum growth occurs between pH 6.0 and 8.0, although growth has been reported between pH 4.8 and 9.3 [10].

*Shigella* spp. are closely related to *E. coli* in their DNA homology and share some biochemical characteristics as well as reactivity to some of the same antibodies, but despite these similarities, their differentiation should be considered clinically significant based, at least in part, on differences in symptoms expressed by infected individuals [7]. *Shigella* spp. are found most frequently in environments of compromised sanitation and poor hygiene, and although the primary route of transmission is by person-to-person contact, shigellosis can occur after the ingestion of focally contaminated water or food [7]. *Shigella* spp. have not been associated with one specific type of food; foods associated with outbreaks of shigellosis have included milk, salads, chicken, shellfish, and other fresh produce served at a wide range of establishments including restaurants, homes, schools, sorority houses, commercial airlines, cruise ships and military mess halls [10]. Approximately 20% of all shigellosis cases in the US are related to international travel (i.e. travelers diarrhea), with S. *sonnei* being the most prevalent and *S. flexneri* being the second most common in developed countries [82]. However, in developing countries, *S. flexneri* and S. *dysenteriae* type 1 are the most common serogroups, with *S.* *dysenteviae* type 1 having been involved in a lengthy epidemic in southern Africa and major epidemics in other parts of Africa, in Asia and in Central America [12]. These epidemics have resulted in high morbidity and mortality rates, especially in malnourished children, immuno-compromised individuals, and the elderly [12].

All *Shigella* serogroups can cause gastrointestinal infections after an incubation period of 12–50 h, after which time individuals experience watery diarrhea in conjunction with fever, fatigue, malaise, and abdominal cramps (Table 1). Although dysentery can be caused by all four *Shigella* serogroups, *S. dysenteriae* type 1 is the most frequent cause of epidemic dysentery and is associated with a particularly severe form of the illness that may be accompanied by other complications including HUS [82].

Twenty-one (32%) of 65 football players and staff developed shigellosis that was associated with consumption of cold sandwiches; the sandwiches were prepared at the airline flight kitchen [83]. Confirmed or probable shigellosis was identified among 240 passengers on 219 flights to 24 states, the District of Columbia, and four countries between September 14 and October 13 [83].

Outbreaks associated with fresh produce have emerged as an important public health concern. On 10 August 1998, the Ontario Ministry of Health was notified of a family of three persons with *S. sonnei* infection who attended a food fair during July 31-August 3 [84]. Laboratory-based surveillance identified 32 additional persons with *S. sonnei* infection who had eaten at a specific kiosk at the fair or at the restaurant that had supplied the kiosk [84]. Foodhandlers at six (75%) of the eight implicated restaurants reported washing parsley before chopping it; usually parsley was chopped in the morning and left at room temperature, sometimes until the end of the day, before it was served to customers [84].

### 2.10. Staphylococcus aureus

*Staphylococcus aureus* are nonmotile, gram-positive cocci that appear singly or in pairs, tetrads, short chains, or characteristic “grapelike” clusters. Staphylococci are facultative anaerobes that, with the exception of *Staphylococcus saccharolyticus* and *Staph. aureus* subsp. *anaerobius*, grow more rapidly under aerobic conditions [7]. *Staphylococcus* spp. are widespread throughout nature and can be found on the skin and skin glands of mammals and birds, in addition to the mouth, blood, mammary glands, and intestinal, genitourinary, and upper respiratory tracts of infected hosts [7]. Outside the body, *Staph. aureus* can survive for long periods of time in a dry state, and have been isolated from air, dust, sewage, and water, making it one of the most resistant non-spore-forming pathogens [5]. In addition to environmental sources of infection, some reported *Staph. aureus* containing foods include ground beef, pork sausage, ground turkey, salmon steaks, oysters, shrimp, cream pies, milk, and delicatessen salads [7].

*Staph. aureus* grow, depending on the strain, at temperatures ranging from 7 to 47.8 °C and produce enterotoxins between 10 and 46 °C but prefer an optimum temperature between 40 and 45 °C. The bacterium grows between pH 4.5 and 9.3, with an optimum between 7.0 and 7.5, and is very tolerant to high levels of salt (>10% sodium chloride); enterotoxin production requires a minimum *aw* of 0.86, whereas growth has been demonstrated at an αw of 0.83 [5],[7],[10].

*Staph. aureus* typically causes infections involving the skin, such as boils, cellulitis, impetigo, and postoperative wound infections, but can also be associated with more serious infections like bacteremia, pneumonia, osteomyelitis, cerebritis, meningitis, and abscesses of muscle, urogenital tract, central nervous system, and various abdominal organs [7]. Toxic shock syndrome, a condition resembling septic shock and resulting from the production of toxic shock syndrome toxin 1, has been attributed to *Staph. aureus* infection [7]. Humans are the major reservoir for *Staph*. *aureus*, and contamination of food can occur through direct contact, indirectly by skin fragments, or through respiratory tract droplets, with most staphylococcal food poisoning cases being traced to food contamination during preparation because of inadequate refrigeration, inadequate cooking or heating, or poor personal hygiene. After ingestion of the enterotoxin and an incubation period of less than 6 and up to 10 h, symptoms may include vomiting, nausea, abdominal cramps, headache, dizziness, chills, perspiration, general weakness, muscular cramping and/or prostration, and diarrhea that may or may not contain blood [7]. The CDC estimates that, in the US, staphylococcal food poisoning causes approximately 241,188 illnesses, 1,064 hospitalizations, and 6 deaths each year [5].

The presence of *Staph. aureus* in food may be considered a public health hazard because of its ability to produce enterotoxin and the risk of subsequent food poisoning. Although there are nine identified staphylococcal enterotoxins, designated as A, B, C1, C2, C3, D, E, F, and G, types A and D are responsible for the majority of the outbreaks [85]. Staphylococcal enterotoxins are included in a larger family of toxins, known as pyrogenic toxins, that have the unique ability to act as superantigens, thereby stimulating an extraordinarily high percentage of T cells. They are difficult to inactivate with heat, because temperatures required to inactivate them are higher than those needed to kill the organism [7]. Staphylococcal enterotoxin A is more heat sensitive than enterotoxins B or C and requires heating at 80 or 100 °C for 180 or 60 s, respectively, to cause a loss in serological reactivity [7].

### 2.11. *Vibrio* spp.

The genus *Vibrio*, belonging to the family Vibrionaceae, contains more than 35 species, of which nearly half have been described in the last 20 years and more than one-third are pathogenic to humans [7]. Organisms in this genus are non-spore-forming, primarily motile, facultatively anaerobic, Gram-negative straight or curved rods. All pathogenic *Vibrio* species, including *Vibrio cholerae, Vibrio parahaemolyticus*, and *Vibrio vulnificus*, require sodium for optimum growth. They are found primarily in brackish or marine environments located in tropical or temperate areas, because their incidence decreases significantly as water temperature falls below 20 °C [7]. *V. cholerae* are also motile by means of a single polar-sheathed flagellum; these curved rods thrive in their environmental reservoir as part of the microflora found in estuaries. In addition to its primary environmental source, *V. cholerae* has been isolated from areas not associated with a marine or brackish water supply, including freshwater lakes and rivers and from birds and herbivores [7]. *Vibrio cholerae* O1 is composed of the classic biogroup that has been isolated during previous pandemics and El Tor, which is the predominant biogroup of the current pandemic [86].

The optimum temperature for growth of *V. cholerae* is between 30 and 37 °C, although growth can occur between 10 and 43 °C. *Vibrio cholerae* grow at pH 5.0–9.6 but prefer a pH of 7.6. They grow at a *aw* of at least 0.97 but prefer 0.984. Optimum growth occurs in an environment with a sodium chloride concentration of 0.5%, although *V. cholerae* growth can occur at concentrations of 0.1–4.0% [10].

*V. cholerae* typically gain entrance into the human body through ingestion of a contaminated food, such as mollusks (raw oysters) or crustaceans eaten raw, undercooked, or even contaminated after cooking, or exposure of an open wound to a contaminated water source. Conditions resulting from *V. cholerae* O1 infection range from asymptomatic to the most severe form known as “cholera gravis” and in part depend on which biogroup is involved, because 75% of the El Tor biogroup and 60%) of the classic biogroup lead to asymptomatic infections [7]. Additionally, the El Tor biogroup results in severe disease in 2% of the infected individuals and mild or moderate disease in 23% whereas the classic biogroup produces severe disease in 11%, of individuals and mild or moderate disease in 30% [87].

After an incubation period of several hours to 5 days, depending on inoculum size and the amount of food ingested, typical symptoms include muscle cramping caused as a result of severe dehydration (fluid loss up to 500–1000 ml/h) resulting from vomiting, increased peristalsis followed by loose stools progressing to watery stools, and mucus-flecked diarrhea that is characteristic of cholera [88]. In addition to dehydration, other complications may include hypovolemic shock, hypoglycemia, and metabolic acidosis [88].

The disease caused by *V. cholerae* O139 Bengal is clinically identical to the symptoms exhibited by *V. cholerae* O1-infected individuals. Other *V. cholerae* serogroups, in addition to *V. cholerae* O1 and *V. cholerae* O139 Bengal, are known as non O1, non agglutinating vibrios or noncholera vibrios and are not known to cause epidemic disease. However, noncholera vibrios are known to cause self-limiting gastroenteritis and also may cause wound infections, bacteremia, and septicemia when associated with a preexisting liver condition [89]. The infectious dose of *V. cholerae* is approximately 10^11^, but with the ingestion of food, the infectious dose is reduced to about 10^6^ depending on the buffering capacity of the food [87].

Food sources implicated as vehicles of transmission for *Vibrio parahaemolyticus* include crabs, prawns, scallops, seaweed, oysters, and clams [7]. *V. parahaemolyticus* grow at temperatures between 5 and 44 °C, with an optimum temperature and pH for growth between 30 and 37 °C and 7.6 and 8.6, respectively; the organism will grow in an environment at pH 4.8–11.0, in sodium chloride concentrations of 0.5–10.0%, and in environments with a minimum aw of 0.94; however, it prefers a concentration of sodium chloride in the range of 2 to 4% and a *αw* of 0.981 [7],[10].

*V. parahaemolyticus* is the *Vibrio* species most frequently isolated from clinical samples obtained in the US [7]. Gastroenteritis is typically associated with consumption of raw, inadequately cooked, or cooked but recontaminated seafood. After a 4 to 96 h incubation period, symptoms of *V. parahaemolyticus* induced gastroenteritis include nausea, vomiting, headache, abdominal cramps, slight fever, chills, and watery diarrhea that is occasionally bloody [7]. Additional symptoms, after exposure to contaminated water, may include infected wounds, eyes, and ears [7].

Although symptoms are usually self-limiting, lasting only 2–3 days, severe cases may result in dysentery, primary septicemia, or cholera-like illness with the possibility of death [87]. The presence of a pre-existing condition (e.g., liver disease, alcoholism, diabetes mellitus, antacid medication, peptic ulcer disease, immune disorder, etc.) greatly enhances the likelihood of developing a clinical syndrome such as gastroenteritis, wound infection, or septicemia [7]. *V. parahaemolyticus* possess four hemolytic components, including a thermostable direct hemolysin (TDH), a thermolabile direct hemolysin, phospholipase A, and lysophospholipase [7]. *V. parahaemolyticus* are invasive and can penetrate the lamina propria and enter circulation, as they have been found in the heart, spleen, pancreas, and liver [85].

During the past two decades in China, *V. parahaemolyticus* has been the most common cause of the bacterial foodborne outbreaks and among the leading causes of bacterial foodborne disease outbreak in many Asian countries, including Japan and India [88]. For the years 2003–2008 *V. parahaemolyticus* gastroenteritis outbreaks in 12 provinces were investigated from China National Foodborne Diseases Surveillance Network. 322 gastroenteritis outbreaks due to *V. parahaemolyticus* were reported, resulting in 9,041 illnesses and 3,948 hospitalizations [89]. A single food commodity was implicated in 187 (58%) outbreaks, of which 58 (31%) involved meat and meat products, and 52 (28%) involved aquatic products [89]. Outbreaks most frequently occurred in restaurants (39%), cafeterias (30%), and private residences (15%); to prevent and control *V. parahaemolyticus* gastroenteritis outbreaks, food workers and consumers should receive training on avoiding cross contamination of ready-to-eat foods with uncooked seafoods, particularly in warm weather months [89].

*V. parahaemolyticus* infection has been considered the leading cause of bacterial illnesses mainly associated with seafood consumption in Guangdong province in China [90]. From 2010 to 2014, 71 outbreaks due to *V. parahaemolyticus* were reported China National Foodborne Diseases Surveillance Network, resulting in 933 illnesses and 117 hospitalizations without death [90]. A food item was implicated if *V. parahaemolyticus* was isolated from food or based on epidemiologic evidence; aquatic products (27 outbreaks, 38.0%), meat and meat products (9 outbreaks, 12.7%), plant-based foods (6 outbreaks, 8.4%), mixed foods (5 outbreaks, 7.0%) were the most commonly implicated foods. Outbreaks most frequently occurred in restaurants (50.7%), private residents (21.1%), and cafeteria (12.7%) [90]. In order to prevent *V. parahaemolyticus* outbreaks caused by cross contamination, improper cooking and improper storage in high-temperature seasons, regulations for seafood safety from the production stage to the consumption stage should be strengthened [90].

Consumption of raw shellfish, primarily oysters, was linked to several multistate *V. parahaemolyticus* illness outbreaks in the US [88]. Animal-based (i.e. meats, such as poultry, internal organs, beef, deli meat and cured meat, and aquatic products, such as crustacean, mollusks and fish) foods were the most common single commodity reported (61%), followed by mixed foods (19%), and other foods (17%) [88].

### 2.12. Yersinia enterocolitica

The genus *Yersinia* belongs to the family *Enterobacteriaceae* and includes 10 established species, although only 3 are considered pathogenic to either humans or animals. *Yersinia pestis* is the causative agent of plague, *Yersinia pseudotuberculosis* is primarily an animal pathogen but may infect humans after the ingestion of contaminated food or water, and *Yersinia enterocolitica* has surfaced as a cause of foodborne gastroenteritis in humans [7],[91]. *Yersinia* spp. are Gram-negative or gram-variable, non-spore-forming rods that grow under both aerobic and anaerobic conditions but are considered facultative anaerobes. With the exception of *Y. pestis*, all *Yersinia* spp. possess peritrichous flagella and are motile at 22–30 °C but not at 37 °C [7].

Twenty-six member states reported 7,202 confirmed cases of yersiniosis in 2015, making it the third most commonly reported zoonosis in the EU [4]. The EU notification rate was 2.20 cases per 100,000 population which was 6.8% higher than in 2014 [4]. There was a statistically significant decreasing 8-year trend in 2008–2015; *Y. enterocolitica* was the most common species reported to be isolated from human cases [4]. The most common serotype was O:3 followed by O:9 and O:5,27. No fatalities were reported among the 4,304 confirmed yersiniosis cases for which this information was reported in 2015 [4].

*Y. enterocolitica* are widely distributed throughout the environment and have been isolated from raw milk, sewage-contaminated water, soil, seafood, humans, and many warm-blooded animals such as poultry and, most importantly, pigs [7]. As a psychrotroph, *Y. enterocolitica* may pose a health hazard in contaminated refrigerated foods, although under refrigeration temperatures the pathogen is usually outgrown by other competing psychrotrophs [92].

*Y. enterocolitica* grow at temperatures between 0 and 45 °C but prefer an optimum temperature between 25 and 30 °C [7]. This psychrotroph can survive alkaline conditions as well as any other gram-negative bacterium but does not survive well in acidic environments, because growth occurs between pH 4.0 and 10.0, with pH 7.6 being optimum [7]. Additionally, *Y. enterocolitica* can grow in the presence of sodium chloride at concentrations as high as 5% [7],[10].

Not all serotypes of *Y. enterocolitica* are enteropathogenic, and the specific serotypes of *Y*. *enterocolitica* involved in hunian yersiniosis are prevalent primarily in swine. Ingestion of contaminated water or food. more specifically raw or undercooked pork, is a source of foodborne infection in humans, resulting in symptoms appearing after an incubation period of a few days to a week. Intestinal yersiniosis may persist for 1–2 weeks in adults and as long as 4 weeks in children and may include symptoms such as watery, sometimes bloody, stools or bloody diarrhea in conjunction with fever, vomiting, and abdominal pain [5],[7]. Immunocompromised individuals and children under the age of 15 are most commonly infected, and extraintestinal infections associated with yersiniosis include septicemia, meningitis, Reiter syndrome, myocarditis, glomerulonephritis, thyroiditis, and erythema nodosum [85],[91]. *Y. enterocolitica* toxin is heat stable, resists enzymatic degradation, remains stable during prolonged storage, and is of similar pH stability as the thermostable enterotoxin produced by ETEC [92].

In July 2011, a cluster of *Y. enterocolitica* infections was detected in southwestern Pennsylvania, US [92]. The outbreak was investigated for the source, in order to prevent further transmission; twenty-two persons were diagnosed with yersiniosis; 16 of whom reported consuming pasteurized dairy products from a local dairy [92]. Because consumption of pasteurized milk is common and outbreaks have the potential to become large, public health interventions such as consumer advisories or closure of the dairy must be implemented quickly to prevent additional cases if epidemiological or laboratory evidence implicates pasteurized milk as the outbreak source [92]. In addition, *Y. enterocolitica* serogroup O:8 was isolated from 24 fecal specimens of 21 patients and 3 kitchen staff in an outbreak in Japan; fresh vegetable salad was confirmed as the incrimination food of this outbreak [93].

## 3. Foodborne Viruses

Viruses are particulate in nature and multiply only in other living cells. Thus, they are incapable of survival for long periods outside the host. More than 100 types of enteric viruses have been shown to cause foodborne illness; the most common foodborne virus pathogens are Hepatitis A and Noroviruses. These viruses are frequently transmitted via food; bivalve molluscs, such as clams, cockles, mussels, and oysters, are especially prone to transmit viruses. The waters in which they grow are increasingly subject to human fecal contamination, sometimes from sewage discharges and sometimes from infected shellfish harvesters. The shellfish collect viruses in the course of their filter feeding activity. Human viruses do not infect these species, but they are harbored for days or weeks in the shellfish digestive tract and are apparently more difficult to remove than bacteria during processes intended to cleanse the shellfish (e.g. depuration) [94],[95]. Unlike many other seafoods, shellfish are usually eaten with their digestive tracts in place. They are often eaten raw or lightly cooked. Shellfish, unlike other foods, may also protect viruses from thermal inactivation during cooking [96].

### 3.1. Hepatitis A

Hepatitis A virus particles are environmentally hardy organisms that can be transmitted by contaminated food, water, environmental surfaces (e.g., contaminated table tops, cooking utensils) and through direct or indirect person-to-person contact [5]. Hepatitis A cannot grow in the environment, however, they are considered to be extremely stable under a wide range of environmental conditions, including freezing, heat, chemicals, and desiccation [5]. Although Hepatitis A share some major characteristics with other genera of the picornavirus family, it is sufficiently different that it is classified as the only species in the genus *Hepatovirus* [97]. There are six Hepatitis A genotypes (I-VI), as determined by RNA sequence analysis. Genotypes I, II, and III contain strains associated with human infections, with the majority of human strains grouped within genotypes I and III. The virus is comprised of single positive-stranded RNA genome of approximately 7.5 kilobases and is a non-enveloped (i.e., no lipid-containing envelope), hydrophobic virus 22 to 30 nm in size [5].

The first recorded outbreak of shellfish-associated viral disease resulted from storing clean oysters in a fecally contaminated harbor while awaiting sale [98],[99]. Over 600 cases of Hepatitis A resulted. More recently, outbreaks of viral gastroenteritis and Hepatitis A have been associated with eating usually uncooked shellfish. A clam-associated outbreak of Hepatitis A in Shanghai may have been the largest recorded outbreak of foodborne disease in history, with 292,301 cases [98]. Sporadic viral illnesses associated with shellfish have also been demonstrated [99]; it is difficult to avoid bias entirely in such studies because, at least in coastal states, a diagnosis of Hepatitis A regularly leads to asking the patient about shellfish consumption, to the exclusion of other foods. Shellfish-growing waters are typically monitored for fecal contamination by testing for bacteria of the fecal coliform group or for *Escherichia coli*. The presence of these bacteria, however, has been shown to be a poor predictor of the presence of human enteric viruses [100]. Unfortunately, no more accurate index of the presence of viruses in shellfish or their growing waters has yet been identified. Because it has no other way to guarantee the safety of raw cockles, the U.K. government allows their sale only if cooked by an approved method.

In 2003, a series of Hepatitis A outbreaks resulted in 1,000 cases of illness across multiple states and 3 deaths. The outbreaks were linked to green onions imported from four farms in Mexico where hepatitis A is endemic and the FDA subsequently banned imports from these farms [101].

The multinational Hepatitis A outbreaks occurring in Europe in 2013 and 2014 with over 1,400 cases linked to fresh and frozen strawberries and berry mix evidenced the usefulness of virus sequencing to link sporadic cases reported in different EU countries in outbreaks [102],[103]. However, due to different sequencing practices and protocols in EU, the interpretation of the sequencing results was often challenging and untimely. Molecular data based on WGS are increasingly replacing the numerous different methodologies currently in use in human and veterinary reference laboratories for outbreak investigation and attribution modeling. These methods have the potential for early identification of foodborne organisms with epidemic character and the resulting data is beginning to be integrated into risk assessment studies. The epidemic potential of a virus genotype or even a subtype, may vary considerably, and is a function of its inherent genetic characteristics and their capacity to mutate, survive and spread through the food chain.

The numbers of reported foodborne illnesses are fewer than actually occur because the CDC's passive data collection system records only illnesses occurring as outbreaks, rather than those occurring sporadically. Hepatitis A, which is notoriously under reported in the US [104], is the only foodborne viral disease in which official reporting is mandatory for all diagnosed cases. Thus, records of the incidence of the other viral diseases are certain to be less accurate.

### 3.2. Noroviruses

Norovirus cause the majority of acute viral gastroenteritis cases worldwide, including an estimated 5.4 million episodes of foodborne illnesses in the US annually [13]. In addition, according to the WHO, Norovirus is nowadays the leading cause of acute gastroenteritis among children less than 5 years of age who seek medical care [105].

Noroviruses are nonenveloped viruses with a diameter of 30–35 nm and a single-stranded RNA genome of approximately 7.5 kb. The viruses are very diverse and are classified into six genogroups of which only three cause infection in humans; within these genogroups, 30 genotypes have been described to date [106]. Recent improvements to diagnostic techniques have allowed researchers to describe the significant contribution of this highly infectious RNA virus to the burden of food-borne illness, particularly as the cause of numerous outbreaks of food-borne disease in community settings such as nursing homes, hospitals, the military, and cruise ships [107],[108].

Fecal-oral spread is the primary mode of transmission. The virus's abilities to withstand a wide range of temperatures (from freezing to 60 °C) and to persist on environmental surfaces and food items contribute to rapid dissemination, particularly via secondary spread (via food handlers or to family members) [108]. Food can be contaminated at the source (*via* contaminated water) or during preparation [108]. Prevention of infection is difficult because these viruses can persist on environmental surfaces and food items. Comparison of Norovirus sequences collected from around the world over the past decade have raised the possibility that pandemic strains of Norovirus are spread through foods sold internationally, or through person-to-person contact when travelers carry the virus [108],[109]. Recent evidence suggests the possibility of animal reservoirs, but direct zoonotic transmission appears to be rare [110].

Cruise ships provide ideal conditions for the introduction and the rapid, global spread of Norovirus infection. Thousands of passengers from different geographic areas are transported in close quarters to multiple destinations around the world. Passengers and crew often disembark at multiple ports throughout the cruise where they can sample the local foods and culture. Cruise ships account for 10% of all reported outbreaks of Norovirus in the US [13]. With the average carrying capacity of a cruise ship now exceeding 2,500 passengers and crew, these outbreaks often affect a large number of people. In 2010, outbreaks of diarrhea and vomiting among passengers and crew on the Celebrity Cruise ship “Mercury” occurred during three consecutive sailings. More than 10–22% of the passengers and 2–4% of the crew fell ill during each trip, resulting in a total of 1,058 cases of illness over the course of a month [111].

An outbreak of Norovirus gastroenteritis that affected as many as 24 players and staff members from 13 National Basketball Association teams were affected with gastroenteritis symptoms was reported [112]. Four of 5 stool specimens from ill players and staff tested positive for Norovirus genogroup II, with the majority of illness occurring during the first week of December 2010; epidemiologic and laboratory evidence strongly suggested that person to person transmission occurred within at least 1 team during this outbreak [112].

In another study, 286 fecal specimens from 88 oyster-associated gastroenteritis outbreaks were examined for the presence of 10 human enteric viruses using antigenic or genetic detection methods in order to determine the prevalence of these infections [113]. All virus-positive patients were over 18 years old. The most common enteric virus in outbreaks (96.6%) and fecal specimens (68.9%) was Norovirus, indicating a high prevalence of Norovirus infection associated with the consumption of raw or under-cooked oysters. Rapid identification of pathogens is important for the development of means for control and prevention. The results of the present study will be useful to establish an efficient approach for the identification of viral pathogens in oyster-associated gastroenteritis in adults [113].

Norovirus outbreaks occur frequently in EU and it can be difficult to establish whether apparently independent outbreaks have the same origin [114]. Six outbreaks linked to frozen raspberries, were investigated separately over a period of 3 months. In one outbreak at a hospital canteen, frozen raspberries was associated with illness by cohort investigation. Bags of raspberries suspected to be the source were positive for genogroup I and II Noroviruses, one typable virus was genotype GI.6 [114]. These molecular investigations showed that the apparently independent outbreaks were the result of one contamination event of frozen raspberries. The contaminated raspberries originated from a single producer in Serbia and were originally not considered to belong to the same batch. The outbreaks led to consultations and mutual visits between producers, investigators and authorities. Further, Danish legislation was changed to make heat-treatment of frozen raspberries compulsory in professional catering establishments [115].

Foodborne viruses cause considerable morbidity and mortality. Controlling them still means relying on good personal and food hygiene, good agricultural practice, post-harvest controls and effective management of human sewage to prevent onward transmission [115]. The role of the asymptomatic food handlers in contributing to Norovirus outbreaks is becoming increasingly clear, with up to one-quarter of outbreaks attributable to them; handwashing with soap and water remains the best method for removing Norovirus from fingers [115]. However, hand sanitizers formulations supplemented with urea and citric acid may be more effective against viruses such as Norovirus [116].

Risk assessment for transmission of emerging viruses through the food chain should include consideration of all means by which food could pose a hazard, that is not just consumption. New technologies have demonstrated the widespread nature of viral contamination in the food chain, but this does not necessarily correlate with the risk of disease. Finally, understanding people's knowledge and behaviour is just as important as understanding virus characteristics and epidemiology when assessing risks of foodborne transmission [114].

## 4. Foodborne Parasites

Parasites are one-celled microorganisms without a rigid cell wall, but with an organized nucleus. They are larger than bacteria. Like viruses, they do not multiple in foods, only in hosts. The transmissible form of these organisms is termed a *cyst*. Parasites are organisms that derive nourishment and protection from other living organisms known as hosts. They may be transmitted from animals to humans, from humans to humans, or from humans to animals. Several parasites have emerged as significant causes of foodborne and waterborne illness. These organisms live and reproduce within the tissues and organs of infected human and animal hosts, and are often excreted in faeces. The most common foodborne parasites are *Cyclospora cayetanensis*, *Toxoplasma gondii* and *Trichinella spiralis*.

In 2015, 156 confirmed trichinellosis and 41 cases of congenital toxoplasmosis were reported in the EU. The EU notification was 0.03 cases per 100,000 population, and decreased by 57.1% compared with 2014 when the highest notification rate was reported since 2010 [4]. Lithuania reported the highest notification rate followed by Romania and Bulgaria. France reported data with 2-year delay, 216 confirmed congenital toxoplasmosis cases in 2014 [4].

The significant burden in low- and middle-income countries where cycles of parasitic infection are highly specific to food sources all over the world has been emphasized [118]. 357 million cases, 33,900 deaths and 2.94 million disability-adjusted life years (DALYs) are due to enteric protozoa of which 67.2 million cases, 5,560 deaths and 492,000 DALYs are attributable to foodborne transmission [118].

### 4.1. Cyclospora cayetanensis

*Cyclosporra cayetanensis* are protozoan parasites, belonging to the family *Eimeriidae*, that inhabit the small intestine, where they spend the intermediary life cycle stages in the cytoplasm of enterocytes and subsequently produce oocysts containing two sporocysts encapsulating four sporozoites [7]. After subsequent shedding of the oocysts, 7–15 days are required for sporulation to occur. 2 genomes of *C. cayetanensis* have been completed up to now according to the data retrieved from NCBI. The median total length of the genome is 44.2991 Mb [12].

*C. cayetanensis* is capable of causing prolonged illness (6 weeks or longer) in both immunocompromised and immunocompetent individuals, with characteristic symptoms including nonbloody diarrhea, nausea, vomiting, anorexia, bloating, abdominal cramping, malaise, fever, and fatigue [7].

Between 1996 and 1998 *C. cayetanensis* was identified as the etiologic agent in several outbreaks in the US and Canada involving raspberries, baby lettuce, and basil. Currently in the US, *C. cayetanensis* is estimated to cause about 15,000 cases of foodborne illness annually [3].

In 1996, 1,465 persons in 20 states, the District of Columbia, and two Canadian provinces became ill after consuming fresh raspberries that were imported from Guatemala and infected with the parasite *C. cayetanensis* [119].

### 4.2. Toxoplasma gondii

*Toxoplasma gondii* is a protozoan parasite member of the phylum *Apicomplexa*, and an obligate intracellular pathogen that is the causal agent of toxoplasmosis in humans. *T. gondii* uses cats as its primary reservoir and any other warm-blooded animal as an intermediate host [7]. The protozoan may be present as tachyzoites, bradyzoites, or sporozoites, which are the three stages of its life cycle. Tachyzoites and bradyzoites occur in body tissues, where the tachyzoites proliferate and destroy infected host cells and the bradyzoites multiply within tissue cysts. Sporozoites are shed, within oocysts, in cat feces where they sporulate after 1–5 days, surviving for months by utilizing their ability to resist disinfectants, freezing, and drying [7]. 17 genomes of *T. gondii* have been completed up to now according to the data retrieved from NCBI. The median total length of the genome is 64.1936 Mb [12].

In humans, *T. gondii* can be acquired in several ways, including the ingestion of contaminated food or water containing the oocyst, contaminated blood transfusion or organ transplantation, transplacental transmission, or accidental tachyzoite inoculation. *T. gondii* infections typically result from the ingestion of cysts in raw or undercooked meat, with fresh pork and beef appearing to be the primary sources [7]. Toxoplasmosis can result from the ingestion of as few as 100 tissue cysts or oocysts, at which time cyst walls rupture, releasing the sporozoites or bradyzoites to move through the intestinal epithelium and circulate throughout the body [7]. Sporozoites and bradyzoites transform into tachyzoites and begin to rapidly multiply intracellularly, and after host cell death, the tachyzoites invade adjacent cells and repeat the reproduction process; these tachyzoites, by means of the host immune response, are forced to transform back into bradyzoites and form cysts in the local tissue, where they can remain throughout the life of the host organism [7]. Toxoplasmosis symptoms include fever, rash, headache, muscle aches and pain, and swelling of the lymph nodes and may persist for more than a month [5].

*T. gondii* is one of the world's most common parasites. Although cats are the only known host in which the parasite can complete its life cycle, this parasite can use almost all warm-blooded vertebrates, including humans, as hosts. *T. gondii* infections are estimated to cause approximately 87,000 illnesses, 4,400 hospitalizations, and 330 deaths each year in the US, making it the second leading cause of foodborne mortality in the US and the third leading cause of food-borne hospitalizations [13]. The most common sources of toxoplasma are undercooked meat, animal feces, and transmission from mother to unborn child. While most people infected with toxoplasma experience no symptoms, unborn children (who contract it from their mothers) and adults with compromised immune systems risk serious side effects. An estimated 22.5% of the US population over the age of 12 has been infected with toxoplasma. For some countries, this figure is as high as 95%.

Of particular concern are women of childbearing age who have not acquired immunity against this parasite since it can be transmitted via placenta to the fetus (congenital toxoplasmosis). The consequences of congenital toxoplasmosis range from mild to severe to fatal and include: mental retardation, seizures, blindness and death [13]. *T. gondii* is highly amenable to experimental manipulation in the laboratory, and serves as a model system for genetic exploration of parasite biology and host-parasite interactions; this organism has been successfully used in transformation studies with genes from the closely related apicomplexan relative *Plasmodium falciparum* [13].

### 4.3. Trichinella spiralis

*Trichinella spirulis* is a parasitic roundworm belonging to the Phylum *Nematoda*, responsible for most human trichinosis infections. Besides humans, *T. spiralis* can infect most carnivorous mammals. 2 genomes of *T. spiralis* have been completed up to now according to the data retrieved from NCBI. The median total length of the genome is 56.7757 Mb [12].

The adult worms are 1.4–1.8 mm in size and are found embedded in the epithelium of the host's small intestine, where females and males mate [7]. Female adults pass larvae into the blood stream and these reach muscle fiber where they encyst; larvae encysted in muscle remain viable for a long time [7]. The symptoms and pathogenicity are mainly due to the migrating and encystment process which cause pain, fever, edema, neurological disorders and even death. Adult nematodes live in the duodenal and jejunal mucosal epithelium, where they can exist for up to 8 weeks before they are expelled; during this transient period, adult female nematodes can release approximately 1,500 larvae into the bloodstream to travel around the body and subsequently enter muscle tissue, where they can survive for several years [7]. In skeletal muscle, larvae develop, mature, and undergo encapsulation in a calcified wall 6–18 months later. Both the larval and the adult stages are passed from the same host. Encysted larvae remain viable for up to 10 years and are freed by the stomach enzymes of the new host after the ingestion of the encysted flesh [7].

Symptoms, after an incubation period of 3–14 days, include nonspecific gastroenteritis, nausea, vomiting, headaches, fever, visual deficiencies, difficulty breathing, chills, night sweating, eosinophilia, myalgia, and circumorbital edema [7]. The nematode can be thermally inactivated, and therefore the USDA recommends cooking pork products to an internal temperature of 76.7 °C [7]. Currently in the US, *T. spiralis* is estimated to cause about 52 cases of foodborne illness annually, with a case fatality rate of 0.003 [3].

## 5. Concluding Remarks

The WHO Foodborne Disease Burden Epidemiology Reference Group provided in 2015 an estimate of global foodborne disease incidence, mortality, and disease burden in terms of DALYs [105]. The global burden of foodborne hazards was 33 million DALYs in 2010; 40% affecting children under 5 years of age. The US CDC estimated that each year roughly 48 million people in the US gets sick, 128,000 are hospitalized, and 3,000 die from foodborne diseases [120]. The number of confirmed cases, hospitalizations and deaths caused by the most common foodborne pathogens reported in the Foodborne Diseases Active Surveillance Network, US, 2015 is shown in Table 2. These major foodborne pathogens also represent an important economic concern; the annual economic impact in the US from health loss alone is estimated as more than $USD 77 billion [122]. A single outbreak from *E. coli* O104 in Germany was estimated to cost more than $USD 3.5 billion in medical costs and a further $USD 304 million was paid by the European Commission for crop losses due to not selling the fresh produce [123]. The economic impact of food safety outbreaks on food businesses has been analysed recently [124].

**Table 2.** Number of cases, hospitalizations and deaths caused by foodborne pathogens reported in the Foodborne Diseases Active Surveillance Network, US, 2015.

Pathogen	No of cases	Hospitalizations (%)	Deaths (%)
*Campylobacter* spp.	6,309	1,065 (17)	11 (0.2)
*Listeria* spp.	116	111 (96)	15 (12.9)
*Salmonella* spp.	7,728	2,074 (27)	32 (0.4)
*Shigella* spp.	2,688	619 (23)	1 (0.0)
Shiga toxin-producing *Escherichia coli* O157	463	180 (39)	3 (0.6)
Shiga toxin-producing *Escherichia coli* non-O157	796	126 (16)	1 (0.1)
*Vibrio* spp.	192	47 (24)	5 (2.6)
*Yersinia* spp.	139	37 (27)	1 (0.7)
Parasites	1,676	272 (16)	8 (0.5)
Total	20,107	4,531	77

After: [121].

Continued surveillance for foodborne disease outbreaks is important to reveal trends in the foods, regions and pathogens associated. In this field, genotype and subtype information from food contaminant strains is required to trace the transmission source, and to characterize and compare strains. The use of WGS as a tool for subtyping foodborne pathogen isolates has considerable potential for improving the detection of foodborne disease outbreaks, rapidly [125]. Furthermore, as suggested by subtyping data, different strains of foodborne pathogens are differently associated with human disease and such differences can be attributed, among others, to the hardy nature of certain strains enabling them to survive and proliferate in food-related environments, or to their increased virulence towards humans [126],[127],[128]. Hence, strain variability data can also facilitate the assessment of the relationships among various characteristics of foodborne pathogens including their virulence, distribution and epidemiology [129].

From the studies reviewed, the foods implicated in foodborne outbreaks are: fish and seafood [70],[88],[89],[90],[94],[95],[96],[113], liver pâtè [24]–[30], chicken products [33],[36], meat and meat products [37],[51],[52],[68],[76], ice cream [71], raw milk [31], rice dishes [15],[16], pasta and pasta salad [18],[19], peanuts [72], flour [61],[62], cold sandwiches [83], fruit juices [35] and fresh produce [49],[50],[53]–[60],[67],[69],[73],[74],[75],[84],[93],[101],[102],[103],[114]. Fresh produce have attracted great attention during the last 20 years, and it seems that there is some weakness of available international networks, as detection and real-time data show [130].

Risk-based food safety approach is significantly different, compared to the classical hazard-based approach, leading to a major shift in thinking about the ways that science and policy-making in food safety should interplay [131]. In this context, a food safety management system is aiming to estimate the risks to human health from food consumption and to identify, select and implement mitigation strategies in order to control and reduce these risks. According to the Codex Alimentarius, risk analysis is a process consisting of three components: risk assessment, risk management and risk communication [132],[133]. It is now generally recognized that the new approach allows for a sharper diagnosis of food safety problems and the identification of effective mitigation strategies to properly deal with them [132].

Foodborne diseases are a global issue, and a unified and joint approach by all countries and the relevant international organizations is a prerequisite for the identification and control of all emerging foodborne problems that threaten human health and international trade [134]. Most foodborne illnesses are preventable despite being complex in their biology, analysis and epidemiology. Certainly, a combination of knowledge and skills across disciplines is required. Public health agencies, regulatory agencies, the food industry and consumers need to make continuous efforts to prevent contamination of foods on the farm, in processing, in restaurants and homes. With suitable food safety education programs for all involved people, numbers of cases of foodborne illnesses could be minimized.
